# SOCIAL PLACES: SHIFTING NEIGHBORHOOD PERCEPTIONS AMONG AGING AMERICANS DURING COVID-19

**DOI:** 10.1093/geroni/igac059.1191

**Published:** 2022-12-20

**Authors:** Gabriella Meltzer, Brendan O'Shea, Michael Esposito, Lindsay Kobayashi, Ashly Westrick, Jessica Finlay

**Affiliations:** Columbia University, New York, New York, United States; University of Michigan, Ann Arbor, Michigan, United States; Washington University in St. Louis, St. Louis, Missouri, United States; University of Michigan, Ann Arbor, Michigan, United States; University of Michigan, Ann Arbor, Michigan, United States; University of Michigan, Ann Arbor, Michigan, United States

## Abstract

This study examines individual and community factors related to older adults’ perceived losses in places to socialize with people of similar and different ages in their neighborhoods during COVID-19. In the 11-month wave of the COVID-19 Coping Study from March-April 2021, responses to perceived availability were “Less,” “About the Same,” or “More.” Most respondents reported less availability in places to socialize with those of similar (68.0%) or different (68.4%) ages. Ordinal logistic regressions showed respondents who lived alone perceived less availability in places to socialize with those of similar or different ages than those living with others (ORs 0.67, 95% CI 0.47, 0.97). Those living in metropolitan compared to non-metropolitan areas also perceived less availability in places to socialize with those of similar ages (OR 0.62, 95% CI 0.39, 0.98). These findings enhance our understanding of COVID-19-related losses in community resources that facilitate healthy aging in place.

